# The full genome sequence of three strains of Jamestown Canyon virus and their pathogenesis in mice or monkeys

**DOI:** 10.1186/1743-422X-8-136

**Published:** 2011-03-24

**Authors:** Richard S Bennett, Jacob T Nelson, Anthony K Gresko, Brian R Murphy, Stephen S Whitehead

**Affiliations:** 1 Laboratory of Infectious Diseases, National Institute of Allergy and Infectious Diseases, National Institutes of Health, Bethesda, MD 20892, USA

## Abstract

**Background:**

Jamestown Canyon virus (JCV), family *Bunyaviridae*, is a mosquito-borne pathogen endemic in the United States and Canada that can cause encephalitis in humans and is considered an emerging threat to public health. The virus is genetically similar to Inkoo virus circulating in Europe, suggesting that much of the northern hemisphere contains JCV or similar variants.

**Results:**

We have completed the sequence of three isolates of JCV collected in geographically diverse locations over a 57 year time span. The nucleotide identity for the three strains is 90, 83, and 85% for the S, M, and L segments respectively whereas the percent identify for the predicted amino acid sequences of the N, NS_S_, M poly, G_N_, NS_M_, G_C_, and L proteins was 97, 91, 94, 98, 91, 94, and 97%, respectively. In Swiss Webster mice, each JCV isolate exhibits low neuroinvasiveness but high infectivity. Two of the three JCV isolates were highly neurovirulent after IC inoculation whereas one isolate, JCV/03/CT, exhibited low neurovirulence. In rhesus monkeys, JCV infection is accompanied by a low-titered viremia, lack of clinical disease, but a robust neutralizing antibody response.

**Conclusions:**

The first complete sequence of JCV is reported for three separate isolates, and a relatively high level of amino acid sequence conservation was observed even for viruses isolated 57 years apart indicating that the virus is in relative evolutionary stasis. JCV is highly infectious for mice and monkeys, and these animals, especially mice, represent useful experimental hosts for further study.

## Background

Jamestown Canyon virus (JCV), family *Bunyaviridae*, is a mosquito-borne pathogen endemic in the United States and Canada and considered an emerging threat to public health [1]. JCV is a member of the California serogroup of viruses in the genus *Orthobunyavirus *and contains three genome segments, small (S), medium (M), and large (L) consisting of a single strand of negative-sense RNA. JCV was first isolated from *Culiseta inornata *mosquitoes collected near Jamestown Canyon, northwest of Boulder, CO [2]. The serogroup contains members found on five continents that include human pathogens such as La Crosse (LACV) and snowshoe hare viruses in North America; Guaroa virus in North and South America; Inkoo and Tahyna viruses in Europe; and Lumbo virus in Africa.

JCV is distributed over a large geographic range, including much of the United States and Canada. This broad range overlaps with other orthobunyaviruses, such as La Crosse, Trivittatus, and snowshoe hare, and raising the possibility for generation of viruses with reassorted genome segments [1,3,4]. The principal vectors for JCV are *Aedes *and *Ochlerotatus *species, with virus isolations made from 26 species of mosquitoes and 3 species of tabanid flies[3,5]. In the US, white-tailed deer are the primary amplifying host, but mule deer, sika deer, moose, caribou, elk and bison can be naturally infected [1,6-9]. Livestock are also susceptible to infection with virus being isolated from lesions on a horse and antibodies detected in both horses and goats [8,10]. It has been suggested that white-tailed deer populations living close to human residents have been responsible for the observed rise in JCV seroprevalence in humans [9]. Seroprevalance among white-tailed deer in North Carolina, the Delmarva peninsula, and Indiana ranges from 18- 82% with seropositivity increasing with age [7,8,11]. Although JCV does not appear to cause disease in adult deer, it has been shown to be teratogenic, with JCV infection during pregnancy resulting in fawns born paralyzed, dead or aborted [12]. Serum cross neutralization studies have suggested JCV, South River virus, and Jerry Slough virus, all endemic to the United States, are antigenically related [3,13,14]. The virus is genetically similar to Inkoo virus circulating in Europe, suggesting much of the northern hemisphere contains JCV or similar variants [15,16].

In humans, JCV infection causes a mild febrile illness that can lead to infection of the central nervous system (CNS) resulting in meningitis and encephalitis. Unlike LACV, which mainly causes serious disease in children, JCV appears to cause disease predominantly in adults [17]. JCV disease is generally associated with headache, fever, neck stiffness, photophobia, nausea, vomiting, and seizures [18,19]. Respiratory involvement has been reported for JCV [12]. Although JCV infection has been confirmed by PCR of a brain biopsy, human isolates of JCV have not been reported [18]. Serological studies of residents of Alaska indicate an overall JCV infection rate of 17.6% [1]. By age 15, 17% of the Alaskan population has been exposed to JCV, and after age 15, seroprevalence increases to 24 - 30% with 25% of the population showing serological evidence of infection with multiple orthobunyaviruses [1]. JCV seropositivity rates in the continental United States range from 3.5-12.9% in New York, 2.5-10% in Wisconsin, 3.0-15% in Indiana, and 27.7% in Michigan [12]. The precise incidence of CNS disease attributable to JCV is unknown, although retrospective analysis of serum collected from patients with CNS disease in New York State between 1966 and 1981 indicated that 41 cases resulted from JCV infection [17]. Orthobunyavirus infection of animals, and most likely humans, during pregnancy has the potential for teratogenicity [20]. It is evident that the majority of JCV infections are subclinical or associated with mild symptoms.

Little is understood about the genetic relationship of JCV isolates from different regions of the United States. We sought to generate complete genome sequences including the previously unreported L segment sequence, with encodes the RNA dependent RNA polymerase (RDRP). Here we describe the complete genome sequence of three JCV isolates and their level of neurovirulence and neuroinvasiveness in Swiss Webster mice. We also describe the first reported infection of non-human primates with JCV that resulted in viremia of short duration in the absence of clinical disease. Since one of the long-term goals of our laboratory is the development of a live-attenuated virus vaccine for one or more members of the California serogroup viruses, the characterization of JCV was seen as an important step in this process. The mouse model we developed will be useful for studying the pathogenesis of the infection in the central nervous system, and the mouse and monkey models will be useful for future testing of attenuated vaccine candidates.

## Results

### Sequence analysis of viral genomes

The complete sequence of three JCV strains isolated from mosquitoes in Colorado or Connecticut over a span of 57 years was determined (Table 1, with accession numbers). This sequence analysis permitted an assessment of the genetic diversity of JCV isolated in different regions of the United States over this period of time. Both original isolates and biologically cloned derivatives were analyzed when possible to permit a comparison of a defined genomic sequence with a virulence phenotype in mice.

**Table 1:** Passage history and geographic location of isolates of Jamestown Canyon viruses

Virus	Strain	Site of isolation	Vector	Passage history^a^	Accession number
JCV/61/CO	61V2235^c^	Colorado	*Culiseta inornata*	MB, Vero 2	n/a^d^
JCV/61/CO-cl^b^				MB, Vero 6	HM007350-2
JCV/03/CT	3573-03	Connecticut	*Ochlerotatus abserratus*	Vero 2	n/a
JCV/03/CT-cl^b^				Vero 6	HM007353-5
JCV/04/CT-cl^b^	3324-04M	Connecticut	*Ochlerotatus canadensis*	Vero 5	HM007356-8

^a^Cell/tissue type followed by number of passages. MB = mouse brain, total number of passages unknown.

^b^Biologically-cloned derivative.

^c^Prototype strain.

^d^Sequence not submitted.

The genome sequence of JCV/61/CO-cl differed from its uncloned parental virus at two loci in the M segment: G1091A, which resulted in a codon change of aspartic acid to asparagine at codon 347, and T2731C, a synonymous change for codon 893. The genome sequence of JCV/03/CT-cl sequence differed from its uncloned parental virus at one locus on the M segment: G629A, which resulted in a codon change of glycine to serine at codon 193. The uncloned parental stock of JCV/04/CT-cl contained a mixture of JCV and West Nile virus, so genetic comparisons or mouse inoculations were not completed with the uncloned stock. However, due to the limited number of available JCV isolates, the cloned stock of JCV/04/CT was not excluded from further analysis.

**Figure 1:**
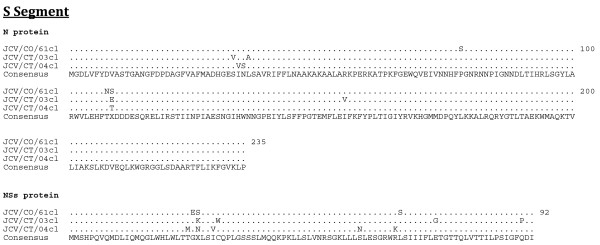
**Amino acid alignment of the predicted coding regions of the S segment**. The consensus sequence consists of two or more sequences sharing the same amino acid residue at a given position and areas of no clear consensus are indicated with an "X".

**Figure 2:**
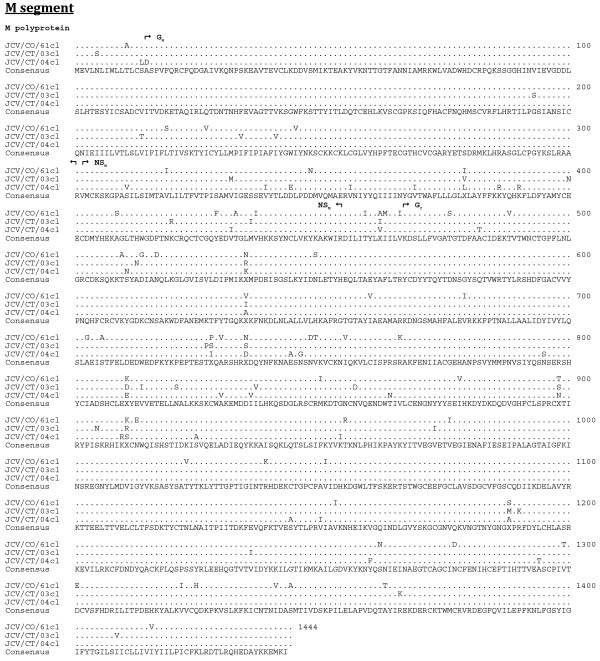
**Amino acid alignment of the predicted coding regions of the M segment**. The consensus sequence consists of two or more sequences sharing the same amino acid residue at a given position and areas of no clear consensus are indicated with an "X".

**Figure 3:**
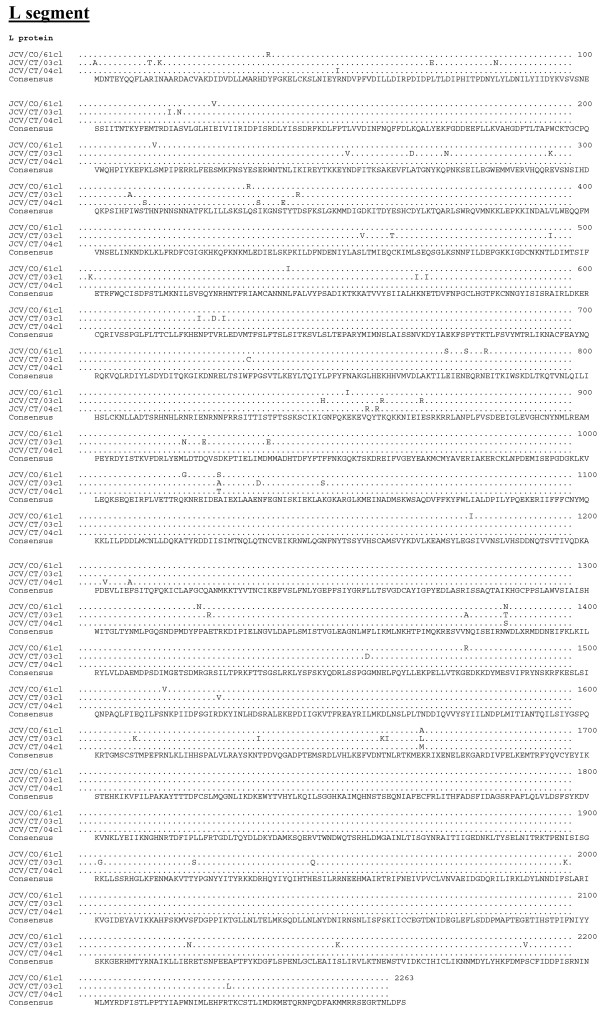
**Amino acid alignment of the predicted coding regions of the L segment**. The consensus sequence consists of two or more sequences sharing the same amino acid residue at a given position and areas of no clear consensus are indicated with an "X".

The length of the respective open reading frames (ORF) of the S, M, and L segments was identical for each JCV isolate, as was the length of the 3' untranslated regions (UTR) (Table 2). However, the length of the 5' UTR differed among the isolates (Table 2). The JCV strains share a minimum nucleotide identity for the S, M, and L segments of 90, 83, and 85%, respectively (Table 3). The predicted respective protein sequence lengths for the N, NS_S_, M polyprotein, G_N_, NS_M_, G_C_, and L proteins were identical for the three JCV strains (Table 4). The minimum percent identity for the predicted amino acid sequences of the N, NS_S_, M polyprotein, G_N_, NS_M_, G_C_, and L proteins were 97, 91, 94, 98, 91, 94, and 97%, respectively. The G_N _protein was the most conserved with less than 2% divergence (Table 4). Complete predicted protein alignments are presented in Figure 1, 2, and 3.

**Table 2:** Segment lengths and UTR/coding region lengths among JCV strains

		Length (nucleotides) for indicated virus		
		
Segment	Region	JCV/61/CO-cl	JCV/03/CT-cl	JCV/04/CT-cl
S	3' UTR	72	72	72
	N ORF	705	705	705
	NSs ORF	276	276	276
	5' UTR	214	209	212
	**Segment total**:	**991**	**986**	**989**
M	3' UTR	52	52	52
	Polyprotein ORF	4332	4332	4332
	5' UTR	126	126	125
	**Segment total**:	**4510**	**4510**	**4509**
L	3' UTR	57	57	57
	L ORF	6789	6789	6789
	5' UTR	114	111	114
	**Segment total**:	**6960**	**6957**	**6960**

**Table 3:** Percent nucleotide identity of the JCV S, M, and L segments amongst the three viruses

	Percent nucleotide identity by segment		
	
Virus pair	S	M	L
JCV/61/CO-cl & JCV/03/CT-cl	91	83	85
JCV/61/CO-cl & JCV/04/CT-cl	90	84	91
JCV/03/CT-cl & JCV/04/CT-cl	92	85	85

**Table 4:** Percent amino acid identity of the JCV predicted proteins amongst the three viruses

	Percent amino acid identity (total protein length in amino acids)						
	
Virus pair	N (235)	NSs (92)	M poly (1444)	G_N_^a ^(286)	NS_M_^b ^(163)	G_C_^c ^(969)	L (2263)
JCV/61/CO-cl & JCV/03/CT-cl	97	93	94	98	91	94	97
JCV/61/CO-cl & JCV/04/CT-cl	98	92	94	99	98	94	99
JCV/03/CT-cl & JCV/04/CT-cl	97	91	96	99	91	96	97

^a ^For this comparison, G_N _is defined as amino acids 16-301 of the M polyprotein.

^b ^For this comparison, NS_M _is defined as amino acids 302-464 of the M polyprotein.

^c ^For this comparison, G_C _is defined as amino acids 476-1444 of the M polyprotein.

**Figure 4:**
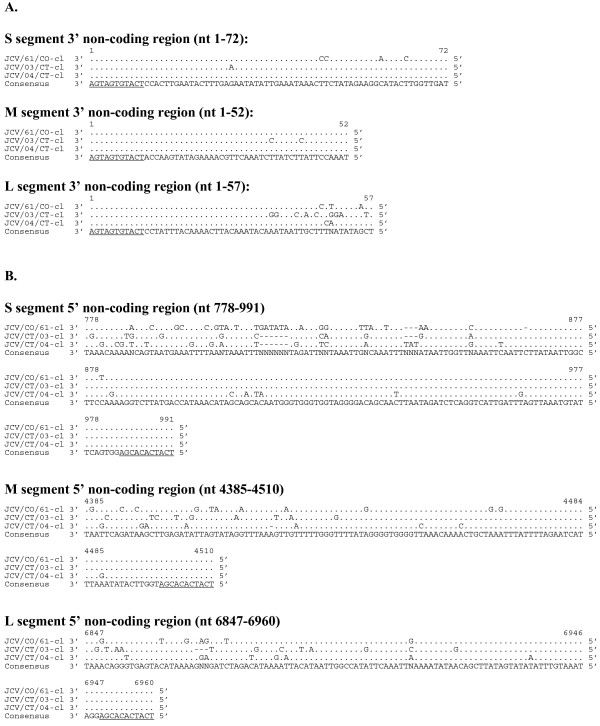
**Alignment of 3' (A) and 5' (B) non-coding regions of the S, M, and L genome segments (cDNA presented)**. For each segment the consensus sequence consists of two or more sequences sharing the same nucleotide at a given position and areas of no clear consensus are indicated with an "N", gaps in the sequences are represented by a dash (-). Underlined sequence indicates region known to be conserved amongst all orthobunyaviruses.

Sequence of the non-coding regions of JCV were more diverse than that observed for the related LACV [21]. The M segment 3' UTR was the most highly conserved with only 2 differences from the consensus (Figure 4A). The 5' UTR was diverse with respect to both length and nucleotide sequence (Table 2, Figure 4B). Within both the 3' and 5' UTR, nucleotide divergence was greatest in the region adjacent to the ORF with the nucleotides in the terminal 50% of the UTRs being highly conserved.

### *In vitro *replication kinetics

**Figure 5:**
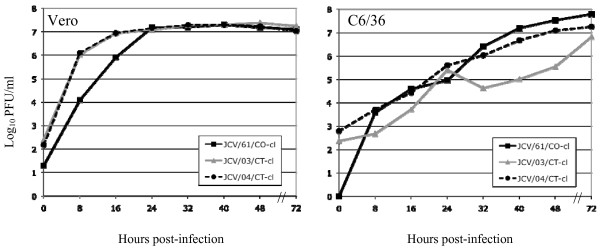
**Growth kinetics of JCV isolates**. Replication of JCV/61/CO-cl, JCV/03/CT-cl, and JCV/04/CT-cl in Vero (monkey kidney) or C6/36 (mosquito) cells infected at a MOI of 0.01.

The kinetics of *in vitro *replication of JCV/61/CO-cl, JCV/03/CT-cl, and JCV/04/CT-cl was compared in Vero and C6/36 cells (Figure 5). Growth kinetics in Vero cells suggest a complete replication cycle is obtained in less than 8 hours with all three isolates reaching peak titers of approximately 10^7 ^PFU/mL within 24 hours. All three isolates replicated more slowly in C6/36 cells with peak titers reached within 72 hours. Cytopathic effects (CPE) associated with JCV infection of Vero cells consisted of cell rounding and detachment from the flask beginning at 24 hours post-infection and complete destruction of the monolayer by 72 hours. CPE was not observed in C6/36 cells infected with JCV.

### JCV pathogenesis in mice

A low level of neuroinvasiveness, LD_50 _greater than 4.0 log_10 _PFU, was observed following intraperitoneal inoculation of weanling mice with any of the five JCV preparations (Table 5). However, the viruses differed in their neurovirulence after IC inoculation, with JCV/61/CO-cl and its uncloned parent virus and JCV/04/CT-cl both being highly neurovirulent, whereas JCV/03/CT-cl and its uncloned parent virus were only minimally neurovirulent. All JCV isolates tested were highly infectious for Swiss Webster mice by both IP and IC routes indicating that the lack of neuroinvasiveness or neurovirulence for any given virus was not due to reduced infectivity (Table 5). Clinical disease in mice infected with either JCV/61/CO-cl (or parent) and JCV/04/CT-cl included lethargy, tremors, seizures, circling, and limb paralysis.

**Table 5:** Jamestown Canyon virus strains are highly infectious for weanling Swiss Webster mice, are weakly neuroinvasive, and differ in neurovirulence

Virus	Neuroinvasiveness^a ^(log_10 _PFU)		Neurovirulence^b ^(log_10 _PFU)		Phenotype^e^	
			
	LD_50_^c^	ID_50_^d^	LD_50_	ID_50_	Neuroinvasive	Neurovirulent
JCV/61/CO	4.8	0.8	1.2	0.5	Low	High
JCV/61/CO-cl	> 4	0.9	0.4	0.4	Low	High
						
JCV/03/CT	> 5	< 0	3.7	0.3	Low	Low
JCV/03/CT-cl	> 5	< 0	> 4	-0.5	Low	Low
						
JCV/04/CT-cl	> 5	0.3	1.4	< -1	Low	High

^a ^Groups of weanling Swiss Webster mice (21-23 days old) were inoculated intraperitoneally with 100 μL of diluted virus (0 - 5 log_10 _PFU).

^b ^Groups of six weanling Swiss Webster mice (21-23 days old) were inoculated intracerebrally with 10 μL of diluted virus (-1 - 4 log_10 _PFU).

^c ^LD_50 _= Lethal dose 50%.

^d ^ID_50 _= Infectious dose 50%. Infection was defined by the development of clinical disease and/or a four-fold increase in neutralizing antibody titers.

^e ^Neuroinvasive phenotype: Low = LD_50 _> 3.5 log_10 _PFU. Neurovirulent phenotype: Low = LD_50 _> 3.5 log_10 _PFU; High = LD_50 _< 1.5 log_10 _PFU.

### Inoculation of rhesus monkeys with JCV/61/CO-cl

To develop a non-human primate model of JCV infection model for testing of future vaccine candidates, rhesus monkeys were inoculated with 10^5 ^PFU of biologically-cloned JCV/61/CO-cl, which was shown to be highly virulent in mice. Illness was not observed in any monkey following subcutaneous inoculation of virus. Viremia was detected in 75% of monkeys with titers ranging from 0.7 - 3.0 log_10 _PFU/mL on day 2 and 1.9 - 2.0 log_10 _PFU/mL on day 4 (Table 6). Virus was not detected in any serum samples collected after day 4. All monkeys developed neutralizing antibody responses against JCV/61/CO-cl with a geometric mean titer of 171 on day 28 post-inoculation. Rhesus immune serum against JCV/61/CO-cl also neutralized JCV/03/CT-cl and JCV/04/CT-cl confirming the antigenic relatedness of all three viruses (Table 6). The GMT neutralizing antibody titer was highest to JCV/04/CT-cl despite primary infection with JCV/61/CO-cl suggesting slight differences in sensitivity of the three strains to neutralization.

**Table 6:** JCV/61/CO-cl infection of rhesus monkeys results in detectable viremia and a high level of neutralizing antibody

	Virus titer on indicated day (log_10 _PFU/mL)^a^		Serum neutralizing antibody titer on day 28 as assayed against indicated virus^b^		
		
Monkey #	2	4	JCV/61/CO-cl	JCV/03/CT-cl	JCV/04/CT-cl
A5E054	0.7	< 0.7	195	64	505
A5E060	< 0.7	< 0.7	253	72	464
A5E068	< 0.7	< 0.7	260	165	571
A5E069	2.2	< 0.7	181	97	201
A5E070	2.5	< 0.7	172	78	205
A5E073	2.5	2.0	166	73	195
A5E077	3.0	< 0.7	180	83	244
DBXG	1.9	1.9	62	18	78
			
		**GMT^c^:**	171	71	259

^a ^Serum was collected on days 0, 2, 4, 5, 8, 10, and 12. Virus was only detectable on days 2 and 4 with a lower limit of detection of 0.7 log_10 _PFU/mL.

^b ^Reciprocal plaque reduction neutralization titer 60% (PRNT_60_).

^c ^Geometric mean titer.

## Conclusions

The first complete sequence of three JCV isolates collected over a 57 year time span from two geographically distant regions is reported. The length of the coding regions for each of the predicted proteins was identical for each of the three viruses as were the length of each 3' UTR. The length of each 5' UTR showed only minor variations. JCV is moderately diverse at the nucleotide level but less so at the amino acid level. There is greater sequence divergence in the areas of 3' and 5' UTR that are adjacent to the ORF and, conversely, greater conservation exists near the termini. The genetic reasons for this difference in sequence conservation were not defined. This genetic diversity in these terminal regions is much more so than described for LACV [21]. The amino acid sequences in the ORFs show only modest sequence divergence with percent identity for the predicted proteins ranging from 91 to 98%. This is relatively modest for viruses isolated 57 years apart in two geographically distinct regions. The modest JCV sequence diversity seen in this study may represent viruses that circulate in separate ecological niches. This theory is supported by Armstrong *et al. *who used sub-regions of the S, M and L segments to identified two major lineages of JCV in Connecticut and demonstrated that their genetic diversity correlated with their geographic location of isolation [22]. One isolate from of each of these lineages was included in this study.

JCV was evaluated for pathogenesis in mice to determine if CNS disease develops with each of the three strains and one virus strain was evaluated in monkeys, which were used as surrogates of the human host. The three strains were highly infectious for mice but were only weakly neuroinvasive following intraperitoneal inoculation. However, the three strains differed in their neurovirulence following inoculation directly into the brain. JCV/04/CT and JCV/61/CO were highly neurovirulent (LD_50 _< 1.4 log_10 _PFU) whereas JCV/03/CT-cl and its uncloned parent virus were only weakly neurovirulent (LD_50 _> 3.6 log_10 _PFU). This phenotype was somewhat surprising since all three JCV isolates replicated equally well in mammalian Vero cells. Since neither JCV/03/CT-cl nor JCV/04/CT-cl were previously adapted to mice, but differed in neurovirulence, it does not appear that passage history was a factor in this phenotype. The genetic basis underlying the difference in neurovirulence between the JCV strains was not further studied.

To evaluate JCV infection in a non-human primate model, we infected JCV seronegative rhesus monkeys with the prototype strain JCV/61/CO-cl. All monkeys were infected, and viremia developed that was detected on days 2 and 4 post-infection and that reached a maximum titer of 3.0 log_10 _PFU/mL. Viremia has not been described in human JCV infections, probably because virus replicates only transiently in the periphery and is cleared before signs of encephalitis become apparent. Infection of rhesus monkeys did not result in any clinical disease, including symptoms of CNS infection, but a robust serum neutralizing antibody response developed in the monkeys. This experience with JCV was similar to that observed with LACV, except that LACV infected monkeys did not develop detectable viremia [23]. It is worth noting that unlike LACV, human isolates of JCV are not available. When such isolates are identified, it would be possible to consider more extensive pathological studies to evaluate the neurovirulence of JCV in non-human primates. Importantly, the three JCV strains evaluated were antigenically similar since infection with JCV/61/CO-cl induced a broad neutralizing antibody response against each of the three JCV strains tested.

The California encephalitis group of viruses, which includes JCV, are responsible for 50 to 167 reported cases of encephalitis per year [24]. Although the majority of these cases are thought to be caused by LACV infection of children, an unknown number are likely to be caused by JCV. However, in a prospective serological study of patients with CNS infections in New York State, evidence of JCV infection was demonstrated in 77% of patients, followed by LACV in 17% of patients, snowshoe hare virus in 4% of patients, and Trivitattus virus in 2% of patients [17]. A vaccine to protect individuals residing in areas of high transmission for LACV or JCV could prevent this severe disease of the CNS. At-risk groups such as constant outdoor workers including forest rangers have a high prevalence of antibodies to JCV [25]. Prior to the introduction of West Nile virus (WNV) in the United States, most encephalitis cases thought to be attributable to an arbovirus infection were not differentiated by the specific virus species. However, new diagnostic tools developed in response to WNV have begun to aid in the determination of virus etiologies for many cases. However, as stated above, the specific contribution of JCV or other California encephalitis group viruses to the overall burden of CNS disease remains largely unknown. Surveillance of mosquito populations for known arboviruses continues to provide clues to the geographical distribution of the viruses and serves to identify regions that may be at higher risk for transmission to humans. Due to overlap in the geographical distribution of many of the California encephalitis group viruses, an effective vaccine would need to be multivalent or cross protective to maximize its usefulness. As a first step in the vaccine development process for JCV and other members of the California encephalitis group of viruses, this paper provides the first full-length genomic sequence of JCV as well as an understanding of the limited genetic diversity among strains and an understanding of their replication and immunogenicity in mice and monkeys. These experimental animals can be used to assess level of attenuation, immunogenicity, and efficacy of live attenuated virus vaccine candidates.

## Methods

### Cells and viruses

C6/36 cells (*Aedes albopictus*) were maintained in Earle's MEM (Invitrogen, Grand Island, NY) supplemented with 10% fetal bovine serum (HyClone, Logan, UT), 2 mM L-glutamine (Invitrogen), and 1 mM non-essential amino acids (Invitrogen). Vero cells (African green monkey kidney) were maintained in OptiPRO™SFM medium (Invitrogen) supplemented with 4 mM L-glutamine. JCV prototype stain JCV/61/CO was obtained from Robert Tesh at the UTMB arbovirus collection. JCV/03/CT and JCV/04/CT were obtained from Philip Armstrong and Theodore G. Andreadis at the Connecticut Agricultural Experiment Station.

### Isolation of biologically-cloned viruses

Biological clones were generated by terminal dilution in Vero cell cultures for JCV/61/CO and JCV/03/CT. Virus stocks were serially diluted in 2-fold increments and inoculated onto 90% confluent monolayers of Vero cells in 96-well plates using eight wells per dilution. After five days of incubation, cell culture fluid was removed to a holding plate, and the cell monolayers were fixed and stained for 10 minutes with crystal violet solution (1% crystal violet in equal volumes of ethanol and methanol). The virus was selected as a clonal derivative when only 1 or 2 of the 8 wells in a single dilution row was positive for JCV CPE. Each virus was terminally diluted three times (sequentially) and amplified in Vero cell culture prior to further analysis.

The parental JCV/04/CT stock contained a mixture of plaque sizes due to an unknown contaminate which produced very large plaques and a medium sized plaque that was recognized by LACV G_N _monoclonal antibody (18752, QED Bioscience, San Diego, CA). The medium-size plaque underwent three rounds of plaque purification and a single round of amplification in Vero cells to obtain a homogenous stock. Plaque purification was performed by infecting confluent monolayers of Vero cells in six-well plates with serial dilutions of JCV/04/CT, and virus was allowed to attach for one hour. Excess inoculum was removed and cells were overlayed with an equal volume mixture of 1.6% SeaPlaque agarose (Cambrex Bioscience, Walkersville, MD) and 2X MEM (Invitrogen) supplemented with 10% FBS and 4 mM L-glutamine. Plaques were allowed to develop for 4 days and overlayed with an additional 2 mL of 1.6% SeaPlaque agarose containing 4% neutral red stock solution (3.3 g neutral red/L PBS, Sigma-Aldrich, St. Louis, MO). After 24 hours of additional incubation, isolated plaques were selected for additional plaque purification and amplification and identified as JCV. The large plaque contaminant in the original parent stock was identified as West Nile virus.

### Sequence analysis of viral genomes

Viral RNA was isolated using the MasterPure RNA Purification Kit (Epicentre Biotechnologies, Madison, WI). Overlapping PCR fragments for the S and M segments were generated using JCV-specific primers and Titan one-step reverse transcription polymerase chain reaction kit (RT-PCR) (Roche, Indianapolis, IN). For the L segment, overlapping PCR fragments were generated using LACV and Tahyna virus-specific primers. Sequencing of the L segment fragments was accomplished by primer walking. Once a full-length draft sequence was assembled, a complete set of sequencing primers was generated and new PCR fragments were re-sequenced. PCR fragments up to 3000 bp were purified and both strands directly sequenced using viral specific primers in BigDye-terminator cycle sequencing reactions analyzed on an ABI 3730 genetic analyzer (Applied Biosystems, Foster City, CA). Sequence data was assembled into a consensus sequence using AutoAssembler 2.1 software (Applied Biosystems).

To sequence the 5' and 3' genome ends of JCV/62/CO, viral RNA was isolated using QIAamp Viral RNA kit (Qiagen, Valencia, CA) from virus-infected cells at 24-48 hours post infection for the 3' untranslated region (UTR) or from clarified cell culture fluid for the 5' UTR. Viral RNA was reverse transcribed using Reverse Transcriptor (Roche) at 55°C with random hexamer primers for the 5' UTR or with genome specific primers at 60-70°C for the 3' UTR that enhanced reverse transcription though RNA secondary structures. cDNA was purified with High Pure PCR product purification kit (Roche), and a poly-A tail was added to the 3' end of the cDNA using 5'/3' RACE Kit, Second Generation (Roche). Genome ends were then amplified using virus and poly-A specific primers. Purified PCR fragments were sequenced as described above.

Once the first and last 13 nucleotides of JCV/61/CO were confirmed to be identical to the known consensus sequence for all orthobunyaviruses, primers that had a known sequence abutted to the 13 nucleotide consensus sequence were generated: primer 1 (forward) 5' gaccatctagcgacctccacagtagtgtact 3' and primer 2 (reverse) 5' gaccatctagcgacctccacagtagtgtgct 3' (underlined sequence corresponds to virus ends). These consensus primers were used to determine the 3' and 5' UTR sequence of all remaining JCV isolates.

### *In vitro *growth kinetics

Cloned JCV strains were used to infect 95% confluent monolayers of C6/36 or Vero cells at a multiplicity of infection of 0.01 and incubated for one hour to allow virus to attach to the cells. Infected monolayers were washed twice with sterile PBS and overlaid with medium. Cultures were incubated at 37°C for 7 days with the exception of C6/36 cells which were incubated at 32°C. Tissue culture fluid (0.45 mL) was collected every 8 hours after infection for the first 2 days and once on day 3, mixed with one-tenth volume of 10X SPG buffer (1X concentration: 218 mM sucrose, 6 mM L-glutamic acid, 3.8 mM dibasic potassium phosphate, pH 7.2), and flash frozen on dry ice. Samples were titrated as described above.

### JCV inoculation of mice

The lethal dose_50 _(LD_50_) of JCV was evaluated in outbred Swiss Webster weanling mice (Taconic Farms, Germantown, NY). All animal experiments were carried out in accordance with the regulations and guidelines of the National Institutes of Health. Three week-old weanling mice (n = 6/dose) were inoculated with serial dilutions of uncloned or biologically-cloned JCV in a volume of 10 μL intracerebrally (IC) or 100 μL intraperitoneally (IP). The mice were anesthetized with isofluorane prior to IC inoculation. All mice were carefully observed twice daily for clinical disease including tremors and limb paralysis. Because clinically moribund mice were humanely euthanized before succumbing to infection, moribundity served as a surrogate for the determination of lethality. For calculations of the mouse infectious dose_50 _(ID_50_) mice were considered JCV infected if they either developed clinical disease or demonstrated a four-fold increase in anti-JCV neutralizing antibody titers (see below for neutralization assay).

### Inoculation of rhesus monkeys

Eight JCV- and LACV-seronegative rhesus macaques of Indian origin were inoculated subcutaneously with 10^5 ^PFU of JCV/61/CO-cl. All monkeys were observed daily for clinical signs of disease. Serum samples were collected and frozen on days -7, 0, 2, 4, 6, 8, 10, 12, 14, 21, 28, and 42 post inoculation for determination of viremia or neutralizing antibody titer.

### Neutralization assay

Neutralizing antibody in mouse and monkey serum was quantified by a plaque reduction neutralization assay. Test sera were heat inactivated (56°C for 30 minutes) and serial 2-fold dilutions beginning at 1:10 were prepared in OptiMEM (Invitrogen) supplemented with 2% FBS, 50 μg/ml gentamicin, and 0.5% human albumin (Talecris Biotherapeutics, Inc., Research Triangle Park, NC). JCV was diluted to a final titer of 500 PFU/mL in the same diluent containing 10% guinea pig complement (Cambrex Bioscience) and was added to equal volumes of the serum dilutions and mixed well. Serum/virus mixture was incubated at 37°C for 30 minutes, added to confluent monolayers of Vero cells, and incubated for 1 hour to allow virus attachment. Cells were overlayed with 1% methylcellulose in OptiMEM and incubated for 5 days at 37°C. After incubation, the overlay was removed, and the monolayers were washed twice with PBS and stained with crystal violet to allow for the enumeration of virus plaques. A 60% plaque-reduction neutralization titer was calculated.

## Competing interests

The authors declare that they have no competing interests.

## Authors' contributions

RSB participated in the study design and planning, performed animal studies, data analysis, and drafted the manuscript. JTN and AKG contributed equally to this project and performed animal studies, sequenced virus isolates, and completed growth curves. BRM and SSW supervised the study and participated in its design and planning. All authors read and approved the final manuscript.
